# Encapsulation of Antifouling Organic Biocides in Poly(lactic acid) Nanoparticles

**DOI:** 10.3390/bioengineering4040081

**Published:** 2017-09-26

**Authors:** Aristotelis Kamtsikakis, Eleni Kavetsou, Konstantina Chronaki, Evangelia Kiosidou, Evangelia Pavlatou, Alexandra Karana, Constantine Papaspyrides, Anastasia Detsi, Antonis Karantonis, Stamatina Vouyiouka

**Affiliations:** 1 Laboratory of Polymer Technology, National Technical University of Athens (NTUA), Zografou Campus, 15780 Athens, Greece; aris_kamtsikakis@hotmail.com (A.K.); konstantina163264@hotmail.gr (K.C.); kp@softlab.ece.ntua.gr (C.P.); 2 Laboratory of Organic Chemistry, NTUA, Zografou Campus, 15780 Athens, Greece; eleni29@hotmail.com (E.K.); adetsi@chemeng.ntua.gr (A.D.); 3 Shipbuilding Technology Laboratory, School of Naval Architecture and Marine Engineering, NTUA, Zografou Campus, 15780 Athens, Greece; eva.kiosidou@gmail.com; 4 Laboratory of General Chemistry, NTUA, Zografou Campus, 15780 Athens, Greece; pavlatou@chemeng.ntua.gr; 5 Department of Wood and Two Pack Coatings, CHROTEX S.A. Hellenic Industry of Paints & Varnishes 19th Km National Road Athens-Corinth, 19300 Aspropyrgos, Greece; karana@chrotex.com; 6 Department of Materials Science and Engineering, School of Chemical Engineering, NTUA, Zografou Campus, 15780 Athens, Greece; antkar@central.ntua.gr

**Keywords:** nanoparticles, PLA, biocides, encapsulation, antifouling, marine applications

## Abstract

The scope of the current research was to assess the feasibility of encapsulating three commercial antifouling compounds, Irgarol 1051, Econea and Zinc pyrithione, in biodegradable poly(lactic acid) (PLA) nanoparticles. The emulsification–solvent evaporation technique was herein utilized to manufacture nanoparticles with a biocide:polymer ratio of 40%. The loaded nanoparticles were analyzed for their size and size distribution, zeta potential, encapsulation efficiency and thermal properties, while the relevant physicochemical characteristics were correlated to biocide–polymer system. In addition, the encapsulation process was scaled up and the prepared nanoparticles were dispersed in a water-based antifouling paint in order to examine the viability of incorporating nanoparticles in such coatings. Metallic specimens were coated with the nanoparticles-containing paint and examined regarding surface morphology.

## 1. Introduction

Biofouling is a naturally occurring dynamic process of surface colonization by marine micro- and macro-organisms. The accumulation of aquatic organisms in water-immersed surfaces is an age-old undesirable phenomenon with severe environmental and economic impacts especially to the shipping industry, as for example an increase in fuel consumption and in maintenance expenditures, as well as reduction of the overall active time of the ship. Antifouling paints are commonly utilized among other technologies to discourage or prevent the growth of fouling organisms [1,2,3,4]. The chemically active antifouling coatings usually contain inorganic components (e.g., Cu_2_O) and organic biocides, such as heterocyclic amines, aromatic halides and carbamates [5], which broaden the antifouling range of the coating by complementing the antifouling properties of the primary inorganic compounds. These organic biocides are most often small, mobile molecules simply dispersed in the wet paint, and can diffuse rapidly in the coating matrix so as to reach the surface and prevent microbial spoilage [6,7,8]. However, this high diffusivity also leads to fast biocide leaching under humid conditions and premature loss of coating protection. As a result, paint formulators use very high concentrations of the biocide in order to maintain the antifouling function for a long time. The use of these excessive amounts has raised significant concerns due to the induced environmental pollution, while it may also cause incompatibility issues with the wet paint, such as macroscopic phase separation [7,8].

The encapsulation of biocides in suitable polymeric micro- and nanocarriers is a challenging and promising practice to restrict the aforementioned leachability issue [3,9,10]: the carrier acts as an intercepting barrier to biocide diffusion enabling controlled release within the coating matrix. In parallel, unstable and prone to degradation compounds are protected from their environment via encapsulation [11,12]. A rather limited number of studies have been published looking into these possibilities of exploiting encapsulation for antifouling applications. Poly(methyl methacrylate) (PMMA) [6,7,8,13,14] and polystyrene (PS) [15,16,17] have been mainly used as non-degradable polymeric carriers and the respective microparticles have been prepared based on solvent evaporation methods (Table 1). The demonstrated results convey optimistic messages about enhancing the antifouling properties of the paints. The relatively recent launch on the market of antifouling products based on encapsulation technologies is also an encouraging reason for further research on this field [18].

The current study differentiates from these previous works on the fact that it focuses on the development of particles in the submicron or nanoscale (1–1000 nm) exhibiting also a biodegradable character. Micro-sized particles are considered to modify at a greater level the properties of the paint compared to nanoparticles (NPs), altering often the morphology and the continuity of the coating depending on the concentration. As a consequence, nanoparticles should be preferred for antifouling applications, similarly to the more beneficial practice of using nanocomposites vs. conventionally filled or reinforced polymeric materials [40]. As far as the biodegradable character of the herein prepared NPs is concerned, it offers the opportunity to manufacture an eco-friendly product by using polymers with an adequately susceptible and adjustable to hydrolysis structure, such as aliphatic polyesters [41]. Therefore, poly(lactic acid) (PLA) was used in the current study; it is a biodegradable and biocompatible aliphatic polyester which has already been utilized in several encapsulation studies [12,42], even for antifouling applications [23], and it is a material clinically tested and approved by the US Food and Drug Administration (FDA) and other regulatory agencies in many countries. Its sufficiently high glass transition temperature (*T*_g_ > 50 °C) fosters its usage as encapsulation carrier of the biocides, while it is considered a hydrophobic material compared to other environmentally friendly biodegradable polymers (e.g., PLGA) making it an advantageous option for marine antifouling applications [42].

As far as the biocides are concerned, Irgarol 1051, Econea and Zinc pyrithione (ZPT) were chosen to be encapsulated. Irgarol 1051 is a symmetrical non-metallic s-triazine with algaecidal properties which has been of a wide concern due to its extensive application, its toxicity and its widespread occurrence in the marine aquatic environment [43,44,45,46]. As a consequence, utilization of Irgarol has been restricted in some countries like UK and Denmark [47]. However, its successful application and the relatively restricted number of alternatives prolong its commercial life while it triggers the need for a more ecologically friendly approach of its usage. Encapsulating Irgarol appears challenging to reduce its concentration in antifouling coatings, while retaining the same antifouling action, by controlling its leachability and avoiding premature degradation of its structure. Econea is a non-metallic organic biocide based on chlorfenapyr [48]. It is considered a promising antifouling compound since it can replace copper due to its wide range of antifouling activity including hard fouling. Econea is a non-persistent compound susceptible to hydrolysis (half-lifetime of 3 h in sea water). However, this feature implies premature degradation and loss of antifouling activity; therefore, encapsulation could enhance its stability in antifouling paints, especially in water-based formulations. Zinc pyrithione (ZPT) is an organometallic salt with numerous applications as an additive ranging from pharmaceutical and cosmetic products to plastics due to its microbicidal properties [48,49]. It is marketed as a non-persistent antifouling biocide which is sensitive to photolysis and rapidly degrades to less toxic compounds. The half-life of ZPT ranges from as low as 15 min to more than 30 d, depending upon the conditions of degradation [50]. ZPT has already been encapsulated successfully using sol-gel technology to silica particles [28], and our interest in encapsulating springs out due its extended application range and its sensitivity to degradation.

Concluding, the objective of the current research was to assess the possibility of encapsulating three commercial biocides in poly(lactic acid) nanoparticles via solvent evaporation technique and characterize the obtained NPs in terms of size, efficiency and thermal properties. A screening attempt to disperse the loaded NPs in a water-based wet paint was also carried out.

## 2. Materials and Methods

### 2.1. Materials

Materials Irgarol 1051 (BASF, Ludwigshafen, Germany), Econea (Janssen PMP, Beerse, Belgium) and Zinc pyrithione (ZPT; Janssen PMP, Beerse, Belgium) were kindly provided by CHROTEX S.A. Commercial PLA (NaturePlast PLI005, Caen, France) was solid state hydrolyzed at 60 °C for five (5) days via pellets suspension in acidic aqueous medium (pH = 3) [51]. The resulting PLA grade was of 135,000 g·mol^−1^ (viscosity-average molecular weight, $\bar{M_{v}}$) and 22.2% (mass fraction crystallinity, χ_c_). Poly(vinyl alcohol) (PVA) (Alfa Aesar, Haverhill, MA, USA) was utilized as an emulsifier, and it was of high molecular weight, 87–89% hydrolyzed. Other reagents used were analytical grade acetone (ACT), dichloromethane (DCM), and acetonitrile (ACN).

### 2.2. Preparation of PLA Nanoparticles (NPs) and Respective Coating

A total of 50 mg of PLA and 20 mg of biocide were dissolved in 5 mL of organic solvent in order to prepare NPs with a 40% biocide:polymer ratio. ACT was used for the encapsulation process of Irgarol 1051 and Econea, while DCM was used for the less soluble in acetone Zinc pyrithione. An oil/water (o/w) emulsion was prepared by sonicating for 6 min in an ice bath (100 W, 30 kHz, 1 cycle, 100% amplitude, Hielscher sonicator UP 100H, Teltow, Germany) the organic phase and 25 mL of 1% (w/v) PVA aqueous solution. After complete evaporation of the organic solvent, the residual dispersions were centrifuged thrice for 20 min (Centrifuge Sorvall RC 28S, ThermoFisher Scientific, Waltham, MA, USA) at 18,500× *g* and 0 °C and washed with deionised water each time. The NP suspensions were frozen and lyophilized overnight in a freeze dryer (Christ Alpha 1–4, Martin Christ Freeze Dryers, Osterode, Germany) without usage of a cryoprotectant. A dispersible in water colorless fluffy mass of NPs was collected after the lyophilization process. The same procedure was followed to prepare unloaded (blank) nanoparticles for comparison reasons.

The yield (Yd) of the encapsulation process was determined by weighting the total amount of the collected nanoparticles in the form of fluffy mass (Equation (1)).

(1) $$Yd=100\times \frac{\left[collected\;mass\;of\;NPs\right]}{\left[initial\;mass\;of\;biocide]+[initial\;mass\;of\;PLA\right]}$$

As a demonstration example, Econea-loaded NPs were prepared via a laboratory 10× scaled-up process. The NPs were then dispersed in a commercially available water-based antifouling paint, and the wet paint was applied on metallic substrates, the surface of which was optically examined using a stereoscope (MZ6, Leica Microsystems, Wetzlar, Germany).

### 2.3. Nanoparticles (NPs) Characterization

#### 2.3.1. SEM Spectroscopy

Scanning electron microscopy was performed on a Jeol 6300 JSM (Jeol USA Inc., Peabody, MA, USA) instrument. The microscope is equipped with a high-sensitivity secondary electron imaging (SEI) detector, and operated at 20 kV on the filament. Samples were coated with an Au coating using a Quorum Technologies SC7620 sputter coater with a sputtering time of 120 s at a current of 10 mA.

#### 2.3.2. Size, Size Distribution and Zeta Potential

The method of dynamic light scattering (DLS), using a Zetasizer Nano ZS instrument equipped with a red He-Ne laser (λ = 633 nm) operating at a backward scattering angle of 173° (Malvern Instruments, Malvern, UK), was utilized to conduct size and size distribution analysis (in intensity mode) of the NPs. The samples were unfiltered aqueous dispersions of the lyophilized nanoparticles with a concentration of 0.1 mg mL^−1^ using ultra-pure water (EASYpureTM UV, Barnstead, Lake Balboa, CA, USA) as the dispersion medium (pH ~ 7). The z-average particle size (PS) and the polydispersity index (PdI) measurements were carried out in triplicate at 25 ± 1 °C. Zeta-potential (ζ-P) measurements were also performed using the same equipment, and determined using a Smoluchovski model. At least, 3 measurements were taken for each sample. As far as the zeta potential is concerned, the same samples were placed in a folded capillary cell (DTS 1060, Malvern, UK) in the Zetasizer Nano ZS instrument; measurements were performed in triplicate at 25 ± 1 °C.

#### 2.3.3. Encapsulation Efficiency (EE) and Preliminary Release Study

The encapsulation efficiency (EE) was determined directly using UV-Vis spectrometry. The amount of each biocide was measured spectrophotometrically (UV-M51, BEL Engineering, Monza, Italy) at 226, 296 and 246 nm for Irgarol, Econea and Zinc pyrithione respectively. The solvents mixture used for the UV-Vis measurements and the calibration curves was a 1:1 v/v water-acetonitrile mixture. 5 mg of the lyophilized NPs were dissolved in 1 mL DCM for 10 min. The resulting solution was left at room temperature until total evaporation of the organic solvent. Appropriate amount of the water-acetonitrile mixture was added to determine directly the encapsulated mass of the biocide and the biocide encapsulation efficiency according to Equation (2):

(2) $$EE=100\times \frac{\left[mass\;of\;encapsulated\;biocide\right]}{\left[mass\;of\;initial\;biocide\right]}$$

Preliminary release studies were made for the case of Irgarol-loaded NPs. Experiments were carried out by suspending 10 mg of loaded NPs into 10 mL of phosphate buffer solution (pH 8) under magnetic stirring at room temperature. At appropriate intervals, the suspension was centrifuged at 12,000 rpm for 20 min. The supernatants were removed after every centrifugation and the precipitated NPs were re-suspended in 10 mL of fresh buffer. The amount of released Irgarol was measured in triplicates using UV-Vis spectroscopy.

#### 2.3.4. FTIR-ATR Spectroscopy

The FTIR-ATR spectra of the antifouling compounds (Irgarol 1051, Econea and Zinc pyrithione), the blank and the loaded PLA NPs were recorded on a JASCO 4200 (JASCO, Gross-Umstadt, Germany) using the ATR technique in the scanning range of 650–4000 cm^−1^.

#### 2.3.5. Thermal Properties

Differential scanning calorimetry (DSC) measurements were conducted in a Mettler Toledo DSC 1 STARe System^®^ (Mettler Toledo, Leicester, UK) through heating the NPs from 25 to 400 °C under nitrogen flow (20 mL·min^−1^) with a rate of 10 °C·min^−1^. The alternating DSC (ADSC) was conducted with a rate of 1 °C·min^−1^ from 90 to 160 °C. Glass transition (*T*_g_) and melting point (*T*_m_) were determined along with the mass fraction crystallinity (χ_c_, %) using Equation (3) for the case of pure PLA samples:

(3) $$\chi_{c}=100\times \frac{\Delta H}{\Delta H_{0}}$$

where, ΔH is the heat of fusion of the sample (J·g^−1^) and ΔH_0_ is the heat of fusion of 100% crystalline polymer (J·g^−1^). For PLLA or PDLA homopolymers, ΔH_0_ is considered equal to 93.1 J·g^−1^ [51].

Τhe DSC measurements were accompanied by thermogravimetric analysis (TGA) measurements performed in a Mettler Toledo TGA/DSC 1 STARe System^®^ (Mettler Toledo, Leicester, UK). The samples were heated from 30 to 600 °C under nitrogen flow (10 mL·min^−1^) with a rate of 10 °C min^−1^. Degradation temperatures (*T*_d_) were determined at the maximum rate of weight loss.

## 3. Results and Discussion

### 3.1. Preparation of PLA Nanoparticles (NPs)—Process Yield

A number of experiments were conducted until reaching an acceptable size (<1000 nm) of particles and a relatively high encapsulation efficiency (EE) value (>75%) in all biocides cases. An overall observation for the pertinent biocide-PLA system, was that NPs size and encapsulation efficiency increased when changing the viscosity of the organic phase either by using a PLA carrier with a higher molecular weight (135,000 vs. 210,000 g·mol^−1^) or by increasing the concentration of the preformed polymer (0.5% vs. 1%). Moreover, the oil/water ratio influenced the particles properties and an increase of this parameter (1:5 vs. 1:10) resulted in a reduction of the size and a minor decrease of EE values. The aforementioned observations are in agreement with other published encapsulation works [52,53,54,55], while the manufacturing process parameters were herein adjusted appropriately and applied as presented in Section 2.2. Accordingly, relatively high NPs yield values (>56%) were determined in all cases (Table 2) except for the comparative blank particles (ca. 30%). This low Yd can be attributed to the low size value of the unloaded particles (Table 3) and thus, to an increase in product loss during the experimental procedure, either in the emulsification or in the centrifugation step.

### 3.2. Size, Size Distribution and Zeta Potential

Particle size and polydispersity index are two of the most significant parameters for the characterization of NP systems, since they shape the release profile of the encapsulated compound, the degradation rate of the carrier, the surface characteristics of the particles, the physicochemical stability of the system and the extent of their effect and interaction when incorporated in a continuous medium [56,57,58,59]. Thus, every application is investigated individually considering the variety of the affecting criteria. More specifically, a system consisting of an antifouling paint and nanoparticles dispersed in the coating requires a fairly homogenous distribution of particle sizes (PS) and an average PS ranging in the nanoscale in order to prevent alteration or deterioration of the coating’s characteristics. However, a small particle size may correspond to rapid release kinetics of the encapsulated biocide or even undesirable “burst release” phenomena [57].

**Figure 1:**
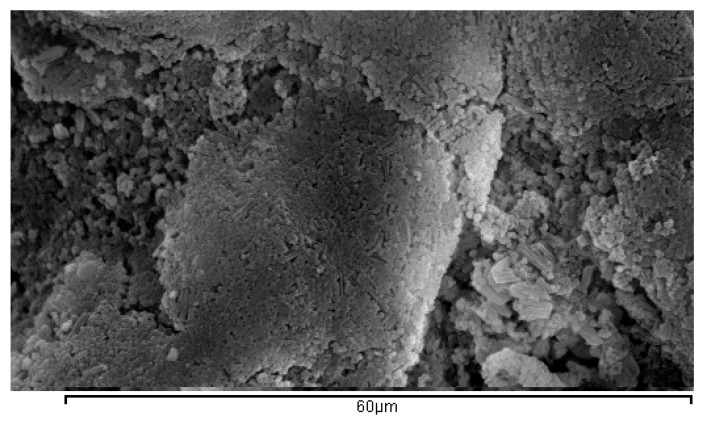
SEM image of Irgarol-loaded poly(lactic acid) (PLA) nanoparticles.

Regarding the morphology of the herein obtained NPs, SEM images (Figure 1) revealed clusters of spherical micro/nanoparticles. Τhe size and size distribution are presented in Table 3 along with the zeta potential values, as measured by DLS method. In particular, all prepared NPs showed z-average size between 311.9 nm and 1013.3 nm and PdI values between 0.169 and 0.592. The PS was found increased in the loaded NPs compared to the blank nanoparticles, as it was also found in the case of a previous work of our group [12]. ZPT-loaded NPs demonstrated the largest PS and PdI values which can be attributed to the different manufacturing process: DCM was used instead of acetone, it is more volatile and may have resulted in increased droplet viscosity during the encapsulation process. On the other hand, the increased PS of Econea-loaded NPs compared to the Irgarol-loaded ones (acetone used in both cases) might be correlated to its greater encapsulation efficiency value (96% vs. 90%). As far as the zeta potential values are concerned, they ranged from −2.17 to −11.33 mV, corresponding to fairly unstable dispersions at neutral pH.

The measured NPs sizes were in the submicron scale, which is in agreement with the objective of this study and can be considered acceptable for the herein suggested application: a well-defined thickness of a painted layer is usually between 50 and 200 μm [3]. Furthermore, in comparison to the previous antifouling encapsulation work [23], in which chlorhexidine-loaded PLA particles of 1 μm were prepared, the particles of this paper exhibit a smaller size when acetone is used as solvent, while particles of similar size are obtained in the case of Zinc pyrithione where DCM is involved in the process. However, it should be noted that a twice as much biocide:polymer ratio is utilized in this work. Thus, the overall preparation procedure of the particles can be considered improved.

### 3.3. Encapsualtion Efficiency (ΕΕ) and Preliminary Release Study

**Figure 2:**
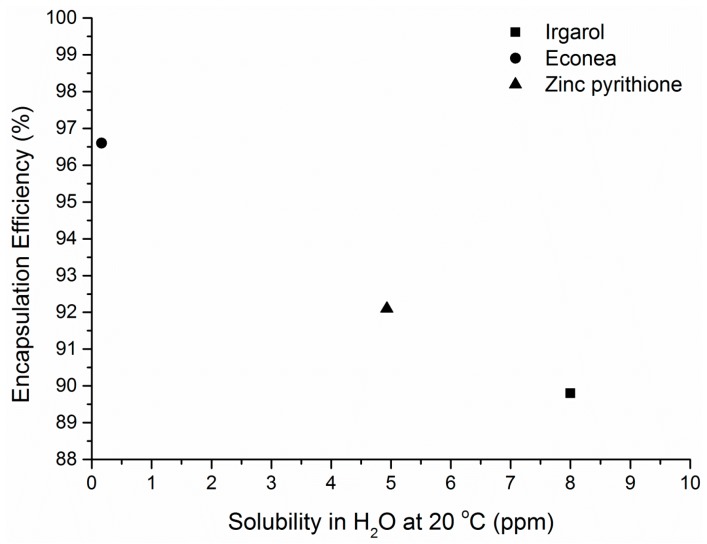
Correlation of encapsulation efficiency and solubility in water of the three tested biocides.

The encapsulation efficiency (EE) is also one of the most critical parameters for estimating the feasibility of the encapsulation process. The obtained values presented in Table 3 are rather encouraging. Significantly high EE values, greater than 90%, were determined directly and may be correlated to the hydrophobic structure of the encapsulated compounds and their low solubility in water (<10 ppm). Econea, which is the least soluble in water, presents the greatest encapsulation efficiency followed by Zinc pyrithione and Irgarol 1051 in a decreasing function between EE and solubility (Figure 2). These findings can be explained when considering the principles of emulsification-solvent evaporation technique: diffusion of compounds from the oil phase to the water phase is less favored with lower water solubility values. As a consequence, antifouling biocides, which are generally hydrophobic and with low water solubility, are expected to be encapsulated successfully with the ο/w emulsification-solvent evaporation technique. This suggestion was also valid in the case of chlorhexidine (800 ppm at 20 °C) which presented an EE value of 85% in PLA microspheres [23].

**Figure 3:**
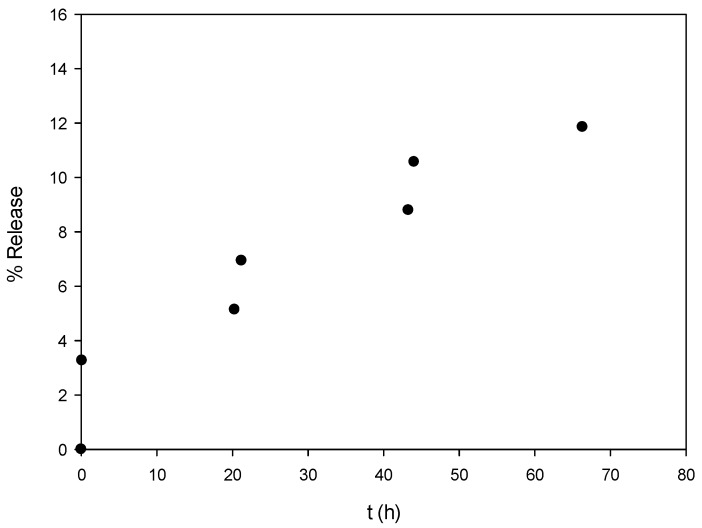
Release profile of Irgarol from loaded PLA nanoparticles.

A preliminary release study was performed with the Irgarol-loaded NPs, which presented the lower particle size. It was found that biocide leaching was lower than 10% for the first 24 h and there was a controlled release profile reaching 12% after 70 h (Figure 3). After 185 h, the % release was increased to 17% presenting however significant error value (±22%). For comparison reasons, it can be mentioned that in the work of Faÿ et al. [23], the release of chlorhexidine from PLA microspheres (average diameter 1 μm, PLA molecular weight 100,000 g·mol^−1^) was found at 39% after 24 h at 20 °C. Further quantification will be carried out sο as to thoroughly investigate the release profiles of the three biocides from the herein prepared PLA nanoparticles.

### 3.4. FTIR-ATR Spectra and Thermal Properties

**Figure 4:**
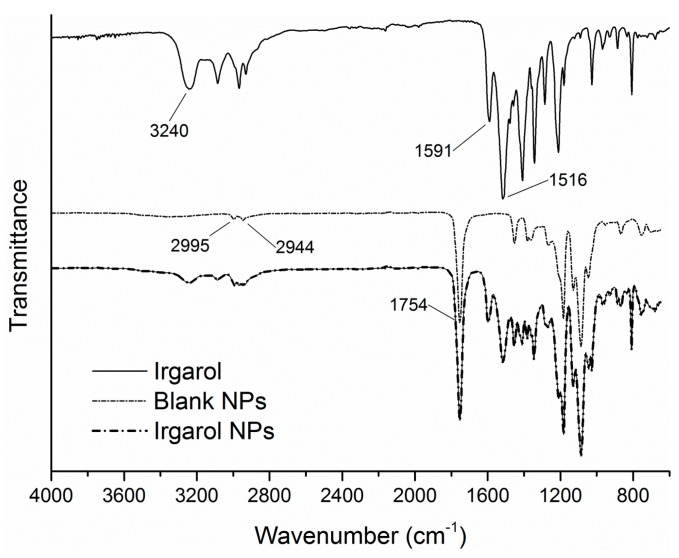
FTIR-ATR spectrum of Irgarol NPs and comparison spectra.

**Figure 5:**
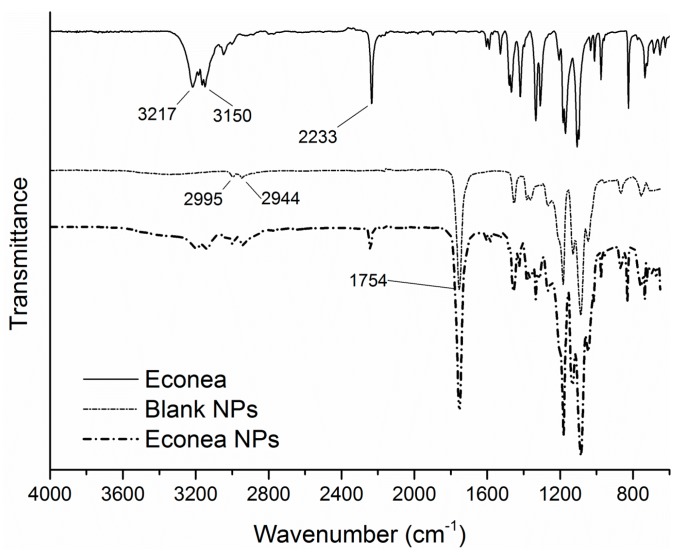
FTIR-ATR spectrum of Econea NPs and comparison spectra.

**Figure 6:**
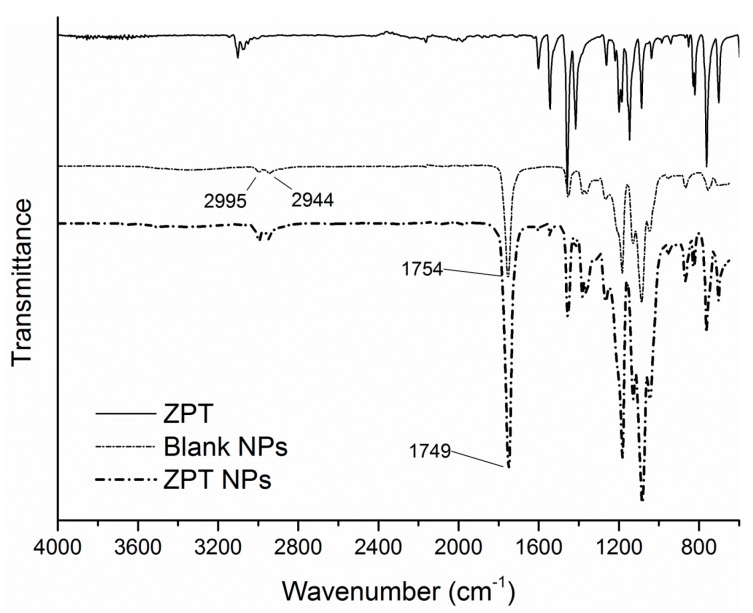
FTIR-ATR spectrum of ZPT NPs and comparison spectra.

In order to obtain an insight of the interaction between the encapsulated biocides and PLA carrier, the FTIR-ATR spectra of the three antifouling compounds, the blank and loaded PLA NPs were obtained (Figure 4, Figure 5 and Figure 6). Starting with the blank NPs, the characteristic peaks of PLA were identified: two peaks at around 3000 cm^−1^ associated to the symmetric stretching vibration of the axial C-H groups from the main polymer chain, along with a very broad peak in the range of 3200–3600 cm^−1^ due to the stretching vibrations of O-H groups. The intense peak originating from ester C=O stretching vibrations was located at around 1754 cm^−1^. Many weaker peaks in the range of 1260–1046 cm^−1^ were assigned to C-O from carboxyl groups and C-O-C (from the ester units) stretching vibrations [41,60]. Turning to the loaded NPs, the characteristic peaks of Irgarol and Econea were obvious in the spectra of loaded NPs implying that biocides are entrapped in the polymer matrix according to nanospheres topology being also present on the surface of the NPs [61]. In particular, for both biocides (Figure 4 and Figure 5), at around 3200 cm^−1^ the characteristic peak due to the N-H stretching vibration appears, and persists in the loaded NPs without a detectable shift, showing that there is no significant interaction between the encapsulated biocide and polymer. The same is valid for the additional peaks of Irgarol at 1591 and 1516 cm^−1^ (probably due to aromatic C=C and C=N bonds) and of Econea at 2233 cm^−1^, which corresponds to the characteristic absorption band for stretching vibration of the nitrile (CN) group. On the other hand, regarding ZPT, the spectrum of the loaded NPs shows mainly PLA matrix absorptions bands (Figure 6): ZPT peaks at ca. 1600 cm^−1^ cannot be clearly seen a fact that probably indicates the absence of the ZPT on the surface of the NPs. In addition, a shift in the wavenumbers of the ester C=O stretching vibration peak of PLA from 1754 cm^−1^ to 1749 cm^−1^ was observed in the spectrum of loaded NPs.

**Figure 7:**
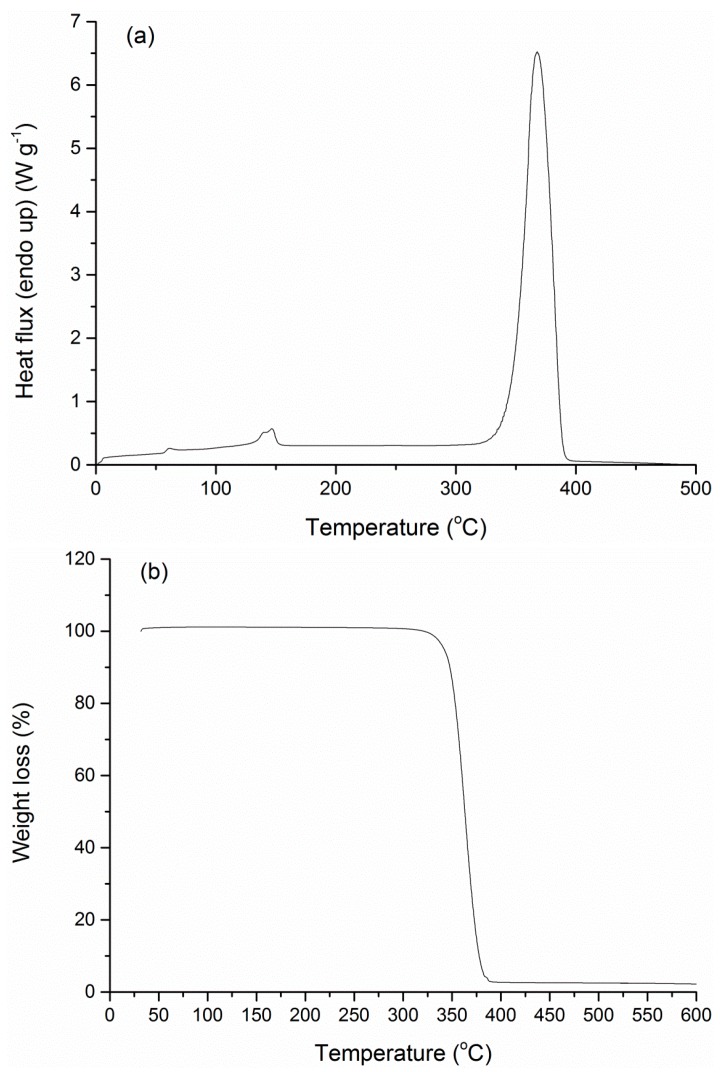
(**a**) Differential scanning calorimetry (DSC) and (**b**) thermogravimetric analysis (TGA) curve of the PLA grade used as encapsulation carrier.

DSC and TGA analysis were further conducted to investigate the thermal properties of the manufactured nanoparticles and to confirm qualitatively the success of the encapsulation process via discernible variations in the thermal curves of the blank and the loaded NPs. Starting with the original PLA grade, the DSC scan revealed a crystallinity of 22.2% (Δ*H*_m_ = 20.6 J·g^−1^) and a glass transition point of 58.8 °C, with a degradation temperature at 365.5 °C (Figure 7).

**Figure 8:**
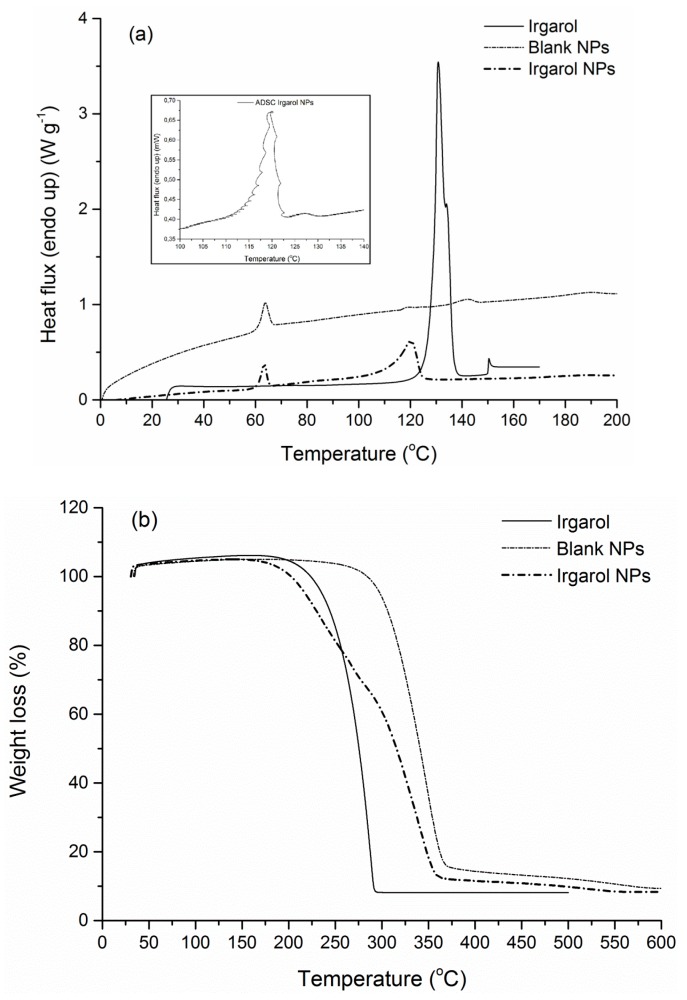
(**a**) DSC and (**b**) TGA curve of Irgarol NPs and comparison curves.

**Figure 9:**
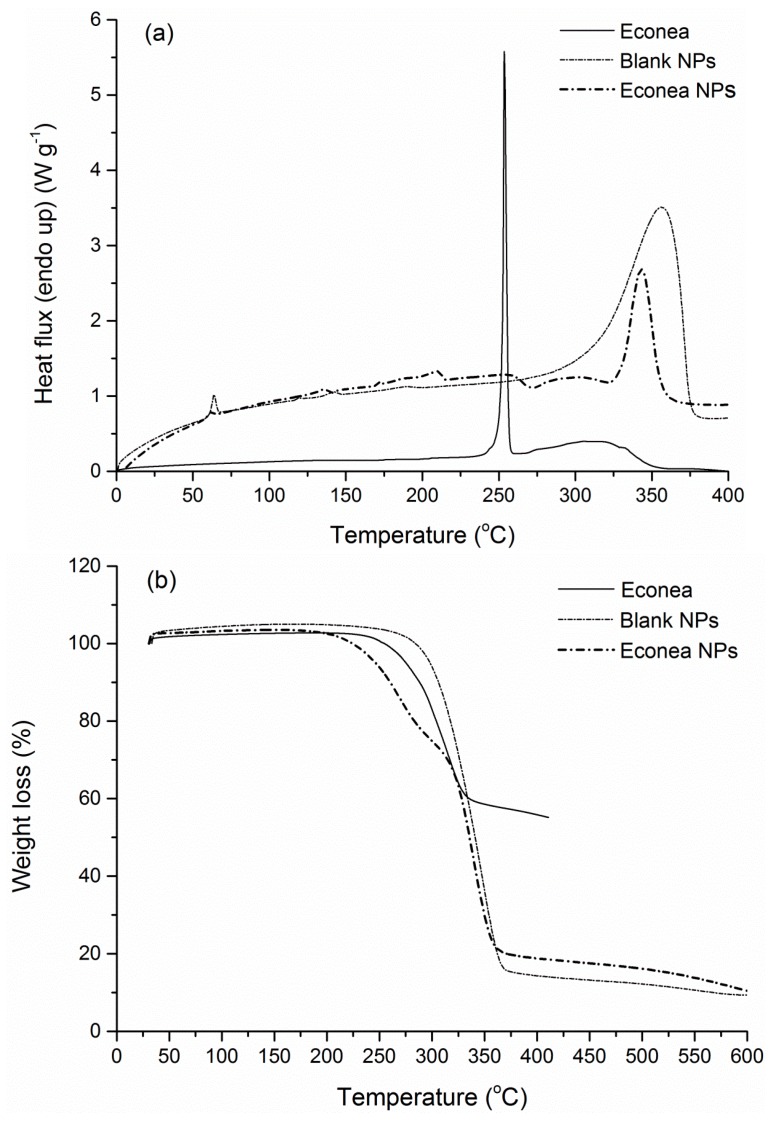
(**a**) DSC and (**b**) TGA curve of Econea NPs and comparison curves.

**Figure 10:**
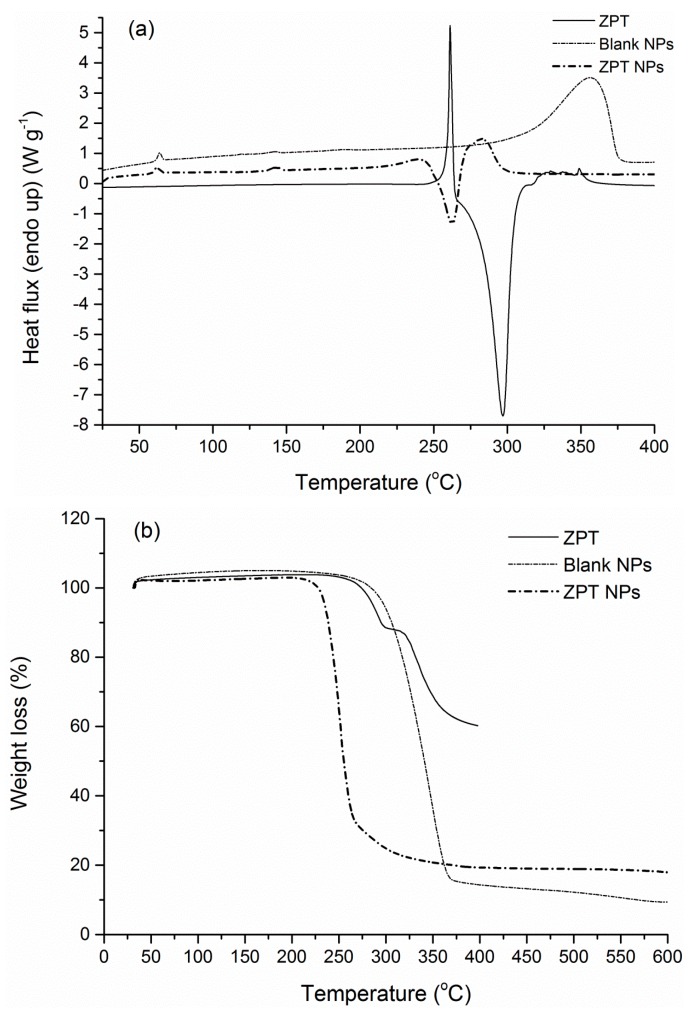
(**a**) DSC and (**b**) TGA curve of ZPT NPs and comparison curves.

Following nanoparticles preparation, both loaded and blank PLA NPs showed negligible crystallinity compared to the polymer matrix and exhibited a behavior of an amorphous material (Figure 8, Figure 9 and Figure 10). Such a low degree of crystallinity corresponds theoretically to increased biocide release and fast degradation of the carrier [62,63]. The low crystallinity in the carrier implies firstly higher amounts of amorphous regions, and thus easier diffusion of the active through the matrix amorphous phase. Especially in the case of biodegradable polymers, such as PLA, the low χ_c_ is also correlated to higher degradation/hydrolysis rates, which in turn facilitate the release of the encapsulated active compound. Furthermore, intense enthalpic relaxation phenomena were detected in the glass transition region of the NPs (55–65 °C) probably attributed to the lyophilization process with the retention of the samples at temperatures lower than the *T*_g_ of the matrix for a period of time. The glass transition point of the NPs was maintained between 57.7–60.1 °C (Table 4), values greater than the destined application temperature range (0–30 °C), rendering PLA an adequate material for the targeted application. Turning to the overall TGA results, an interesting observation is that in all NPs cases, degradation temperatures were found at lower values (251.2–348.2 °C) compared to the polymer matrix (365.5 °C), even for blank NPs where no chemical composition difference exists. This can be attributed to the size of the manufactured particles, since properties of a polymeric material related to surface effects and/or chain confinement and mobility, have been found considerably different for a bulk and a nano-sized sample [64].

Irgarol NPs presented a noticeable endothermic peak near the melting point of the pure biocide (130.7 °C) and the first melting endotherm of the blank NPs (118.7 °C) (Figure 8a). After conducting an alternating DSC analysis (ADSC) in this particular thermal region (110–135 °C), it was observed that the peak was a combination of a prevailing PLA melting peak and a secondary Irgarol peak. As far as the corresponding TGA curve is concerned (Figure 8b), Irgarol NPs were degraded at lower temperatures, via a two-stage degradation mechanism, compared to the single-stage degradation of the blank NPs. These thermal differences can be attributed to the presence of Irgarol which is degraded at 286.2 °C.

Similar is the DSC scan of Econea NPs, where the two small endotherms (135.6 and 210.2 °C) can be attributed to PLA and biocide respectively (Figure 9a). Econea NPs also presented a two-stage degradation profile shifted to slightly lower temperatures due to the presence of the encapsulated compound which is degraded earlier (314.2 °C) (Figure 9b). Moreover, the success of the encapsulation process can be confirmed by the determined residue of the loaded NPs (10.5%), which lies between the two reference values, i.e., it is slightly higher than the blank NPs (9.7%) and lower than the pure compound (55.3%).

Zinc pyrithione NPs presented even higher residue value (17.5%) which can be expected due to the presence of zinc in the structure of the encapsulated biocide (Figure 10b). Degradation phenomena were even more intense with a shorter thermal window compared to all the other cases; degradation temperature was found at 251.2 °C. This behavior might be attributed to the action of zinc as a catalyst of the degradation of the matrix; in the work of Abe et al., the non-isothermal degradation of PLA samples was found dependent on the amounts of residual zinc compounds from the synthesis process [65]. Insignificant crystallinity (<1%) similar to the blank NPs was detected in the same thermal region (Figure 10a).

### 3.5. Coating Based on Nanoparticles

Due to the higher yield (83%) and encapsulation efficiency achieved (96%), Econea was used as a demonstration example to assess the feasibility of the process scaling-up of loaded NPs incorporation into a wet paint formulation. Econea-loaded nanoparticles were successfully reproduced following the same manufacturing technique at a higher laboratory scale (450 mg), resulting in yield value of 70%: the average size of the NPs was determined at 452.9 ± 9.2 nm, the PdI value at 0.401 ± 0.032 and EE at 92%.

**Figure 11:**
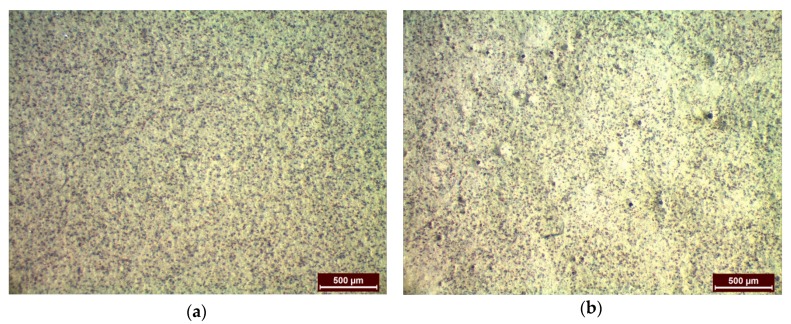
Stereoscopical view of the coated specimens (**a**) without NPs and (**b**) with dispersed Econea-loaded NPs.

An amount of the formed NPs was further dispersed in a commercially available water-based antifouling paint at a concentration of 2% w/v. Metallic specimens were uniformly coated either with pure or with loaded NPs-containing-paint and presented similar appearance in the macroscopic scale. However, stereoscopical observation of the samples revealed differences on the surface of the specimens. Particularly, small holes were easily detectable in NP-containing painted samples which were absent in the comparative NP-free specimens (Figure 11). The size of the holes ranged between 20 and 40 μm. The latter is anticipated to increase the surface micro-porosity and this behavior might be correlated to various factors such as the PdI value of the particles, the dispersion method of the lyophilized NPs or even the selected application method of the paint on the metallic substrate. According to Trojer et al. [3], the porosity of the coating is greatly influenced by the corresponding drying conditions (temperature, drying time). In this case, the drying conditions were common for all coated specimens either coated with NP-free or with NP-containing paint. The absence of surface porosity in the NP-free specimens results in the conclusion that the drying conditions are not the determining factor for the presence of small holes in the NP-reinforced specimens. On the other hand, attempts can be focused on changing the mixing process of the NPs and the paint formulation so as to avoid aggregation phenomena. Further quantification of the herein prepared encapsulation systems will be carried out in order to examine the efficiency of the relevant protective coatings through studying the release of the encapsulated biocides within the coating matrix as well.

## 4. Conclusions

Nanoparticles with antifouling content were successfully manufactured with the emulsification-solvent evaporation technique. The hydrophobic compounds of Irgarol 1051, Econea and Zinc pyrithione were encapsulated with minimal losses in biodegradable poly(lactic acid) nanoparticles reaching yields and encapsulation efficiency values greater than 56% and 90% respectively. The z-average sizes ranged between 312 nm and 1013 nm, while preliminary release studies showed a controlled diffusion profile of the biocide from the nanoparticles. The manufactured NPs presented insignificant crystallinity and their determined glass transition point ensured thermal stability at the application temperature. An almost 10× laboratory scale-up encapsulation process was also successfully conducted for the entrapment of Econea, used as a demonstration example. The yielded NPs were easily dispersed in a commercial water-based antifouling paint; the applied mixing process resulted in a macroscopically uniform appearance of painted metallic specimens, although a more detailed microscopic observation revealed the presence of μ-sized pores on the surface.

## Figures and Tables

**Table 1:** Previous encapsulation studies on antifouling (AF) agents.

Publish Year	Carrier	AF Agent	Encapsulation Technique	Reference
1992	Metallic (Cu) microtubules	*Renilla* extract	Introduction of dry microcylinder powder in saturated solution of the AF agent	[19]
2001	Silica, zeolites	Isothiazolinones (e.g., OIT)	Adsorption of the biocides on the surface of the siliceous frameworks	[20]
2002	Silicates	α-chymotryspin	Two-step polymerization process	[21]
2004	Polystyrene	Zosteric acid	Emulsification–solvent evaporation	[15]
2005	Polystyrene-divinyl benzene beads	Triclosan, phosphonium salts	Dispersion polymerization	[22]
2007	Poly(methyl methacrylate-*co*-butyl acrylate)	4,5-dichloro-2-octyl-4-isothiazolin-3-one (DCOIT)	Two-stage miniemulsion polymerization	[13]
2008	Poly(lactic acid)	Chlorhexidine	Emulsification–solvent evaporation	[23]
2010	Polyethylenimine-Silica	Hexose oxidase	Co-precipitation	[24]
2010	Poly(methyl methacrylate)	Medetomidine	Emulsification–solvent evaporation	[6]
2010	Poly(methyl methacrylate)	4-nitroanisole	Emulsification–solvent evaporation	[14]
2010	Silica	3-iodoprop-2-ynyl *N*-butylcarbamate (IPBC)	Emulsification and cross-linking	[25]
2011	Various polymer layers	DCOIT	Emulsification and cross-linking of the shell	[26]
2011	Poly(methyl methacrylate)	IPBC	Emulsification–solvent evaporation	[7]
2011	Natural polymer (N/A)	Ag compound	Emulsion polymerization	[27]
2011	Silica gel	Zinc pyrithione	Sol-gel technology—Production of aerogels	[28]
2013	Chitosan	Paeonol	Emulsification and ionic gelation	[29]
2013	Polyethyleneimine	Sodium benzoate	Interfacial polyaddition	[30]
2014	Poly(methyl methacrylate)	OIT	Internal phase separation	[8]
2014	Polysaccharide complex of chitosan and xanthan gum	DCOIT	Simultaneous emulsification and cross-linking via ultrasonication	[31]
2014	Polystyrene	IPBC	Emulsification–solvent evaporation	[16]
2014	Polystyrene–polycaprolactone blends	IPBC	Emulsification–solvent evaporation	[17]
2014	Gelatin-urea-formaldehyde	Ag nanoparticles	Dispersion polymerization	[32]
2015	Silica	2-mercaptobenzothiazole (MBT), DCOIT	Emulsification and silica precursor (TEOS) polycondensation	[33]
2015	Carbon	Ag ions	Hydrothermal treatment	[34]
2015	Layered double hydroxides	Cinnamate anions	Acid-salt treatment and ion exchange	[35]
2015	Silica	Bienzyme system	Biomimetic silicification	[36]
2017	Silica	Copper and zinc pyrithione	Emulsification and TEOS polycondensation	[37]
2017	Polyimide	Cu nanoparticles	Solution precursor flame spray	[38]
2017	Halloysite nanotubes	TCPM	Physical entrapment with pressure cycles	[39]

**Table 2:** Measured yield values (Yd) of the encapsulation process agents.

Encapsulated Biocide		Collected Mass (mg)	Yield (%)
Blank NPs		15.6	30
Irgarol NPs	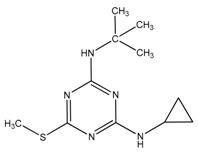	41.0	56
Econea NPs	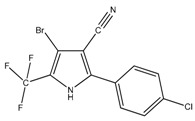	59.6	83
ZPT NPs	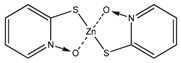	48.3	72

**Table 3:** Size (PS), size distribution (PdI), zeta potential (ζ-P) and encapsulation efficiency (EE) values of the prepared PLA particles.

Samples	PS (nm)	PdI	ζ-P (mV)	Direct EE (%)
Blank NPs	311.9 ± 7.5	0.169 ± 0.015	−11.10 ± 0.46	
Irgarol NPs	465.0 ± 11.9	0.400 ± 0.039	−9.43 ± 0.43	90
Econea NPs	529.2 ± 3.5	0.300 ± 0.009	−2.17 ± 0.11	96
ZPT NPs	1013.3 ± 53.5	0.592 ± 0.008	−11.33 ± 0.57	92

**Table 4:** Thermal properties of PLA, biocides and prepared nanoparticles (NPs).

	*T*_g_ (°C)	*T*_endo_ (°C)	Δ*H*_endo_ (J·g^−1^)	*T*_d_ (°C)	Residue (%)
**PLA**	58.8	146.7	20.6	365.5	2.5
**Blank NPs**	60.1	118.7, 142.0	0.32, 1.6	348.2	9.7
**Irgarol**		130.7	107.1	286.2	8.2
**Irgarol NPs**	59.1	120.3	n.d	232.8, 339.5	8.5
**Econea**		253.5	98.8	314.2	55.3
**Econea NPs**	57.7	135.6, 210.2	n.d	270.6, 338.8	10.5
**ZPT**				289.5, 335.6	60.2
**ZPT NPs**	59.6	143.2	n.d	251.2	17.5
